# Supplementary material for: Strong geometry dependence of the Casimir force between interpenetrated rectangular gratings
Source: Nat Commun. 2021 Jan 26;12:600. doi: 10.1038/s41467-021-20891-4 (PMC7838308; doi:10.1038/s41467-021-20891-4)
Supplement: Supplementary file 1 — Supplementary Information [file 41467_2021_20891_MOESM1_ESM.pdf]

Supplementary information:

Strong geometry dependence of the Casimir force between interpenetrated rectangular gratings

Wang *et al.*

Supplementary Note 1. The boundary of gratings from electron micrographs

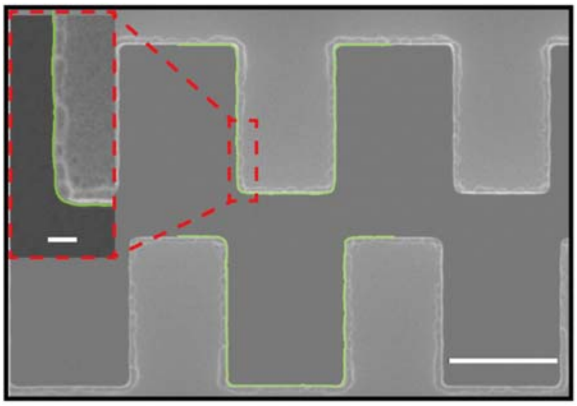

Supplementary Figure S1 Digitalized boundary from the top-view of scanning electron micrographs. Green lines are the digitalized boundary from the micrograph. The inset shows the zoom-in of a portion of the boundary. The scale bar in the main figure measures 1 μm (the width of the grating finger *w* is ~ 900 nm). In the inset, the scale bar measures 100 nm. All simulations on the real device, including SCUFF-EM, PFA, and PAA, are based on the digitalized boundary where the slightly rounded corners are taken into consideration.

Supplementary Note 2. Dependence of Casimir force/force gradient on different grating parameters for gratings made of perfect metal

As discussed in the main text, when two rectangular gratings interpenetrate, the force undergoes an abrupt jump under the PFA (black line in Fig. 1c of the main text). The jump gives rise to a delta function in the force gradient. In contrast, the actual Casimir force, as calculated by SCUFF-EM, increases smoothly (red line Fig. 1c of the main text) and the force gradient displays a peak of finite height and non-zero width. Here we analyze the dependence of constant Casimir force after interpenetration and peak height on the lateral separation g between adjacent grating fingers made of perfect metal using numerical calculations.

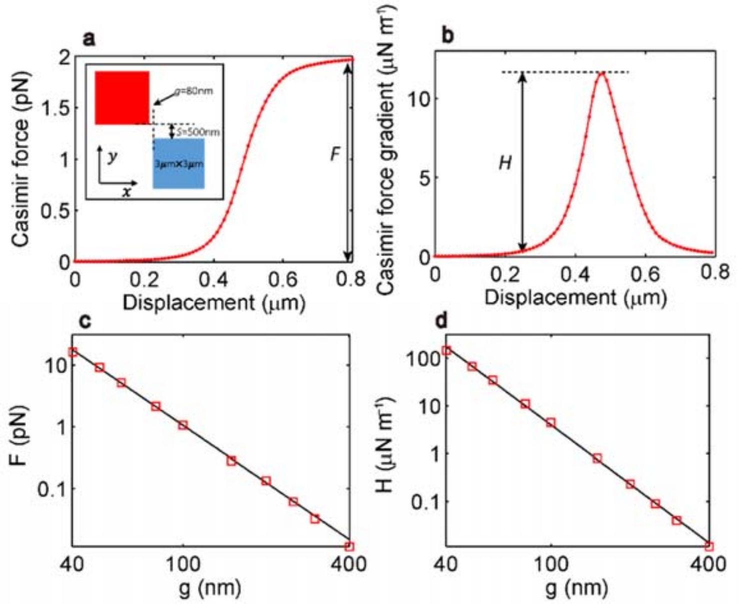

Supplementary Figure S2 Casimir force between two perfect-metal corners, see inset in a: two blocks initially separated by a gap of 80 nm (x-direction) and 500 nm apart in the y-direction. Main figures **a** and **b**: calculated Casimir force and force gradient as a function of displacement in the y-direction. **c** Log-log plot of the constant Casimir force after interpenetration as a function of g. The red squares are the calculated results. The straight line shows that $F \propto 1/g^{3.08}$. **d** Log-log plot of the dependence of the height of peaks on g. The black straight line shows that $H \propto 1/g^{4.11}$

To simplify the calculation, we consider two objects, each with cross-section of 3 μm by 3 μm square as shown in the inset of Supplementary Figure S2a and with thickness of 2.58 μm in the z-direction. The thickness is much larger than the size of the lateral gap (~ 80 nm). The distance between them in the x-direction is kept constant at g = 80 nm while the initial separation in the y-direction is s = 500 nm. Supplementary Figure S2a plots the Casimir force in the y-direction calculated using SCUFF-EM as the lower block is displaced in the positive y-direction until the Casimir force reaches a constant value F. Both blocks are assumed to be perfect conductors. Supplementary Figure S2b shows that the force gradient exhibits a peak with height H.

Next, we repeat the above procedure for the lateral separation g ranging from 40 nm to 400 nm and find the dependence of F and H on g. In Supplementary Figure S2c, we find that F has an inverse power law dependence on g. The solid line in the log-log plot represents a linear fit, with slope -3.08. This result is consistent with the simple argument on perfect metal using PFA in the main text that yields $F \propto 1/g^3$. If the gratings are made of materials with finite conductivity, it is expected that for small g the scaling will change to $1/g^2$ in the non-retarded limit. Supplementary Figure S2d plots H versus g in logarithmic scales. The solid line is a linear fit showing that H has a power-law dependence on g with exponent -4.11.

Supplementary Note 3. Device fabrication

The device is fabricated from a silicon-on-insulator wafer with a p-doped device layer of ≈ 2.58 μm thickness (Fig. S3). The buried oxide layer underneath is ≈ 2.5 μm in thickness. We first grow a layer of thermal oxide (550 nm) as the first mask layer, and deposit a polysilicon layer (200 nm) on top of it as the second mask layer. Afterwards, we use electron-beam lithography to define the gratings which are the most critical part of the device. Electron beam lithography is essential to reduce the rounding of the corners of the rectangular grating and to ensure that the grating units are uniform and accurately defined (up to ~ 5 nm). The pattern on the electron-beam resist is transferred to the polysilicon mask by reactive ion etching (RIE). We then repeat the same process but with ultraviolet stepper lithography to define the other larger structures, such as the comb drive and the springs, that only needs to be accurately defined to ~ 0.5 μm. At the end of this step, the entire device is defined on the polysilicon mask. Next, we transfer the pattern from the polysilicon mask to the oxide mask using RIE and further transfer it to the device layer by deep reactive ion etching (DRIE). To produce near-vertical sidewalls with no undulations, a continuous

etch and passivation recipe is used. Afterward, an oxygen plasma etch removes the hydrocarbon generated by the DRIE. At last, we define the bonding pad for electric connections by optical lithography and evaporate chromium (5 nm) and gold (150 nm) on it.

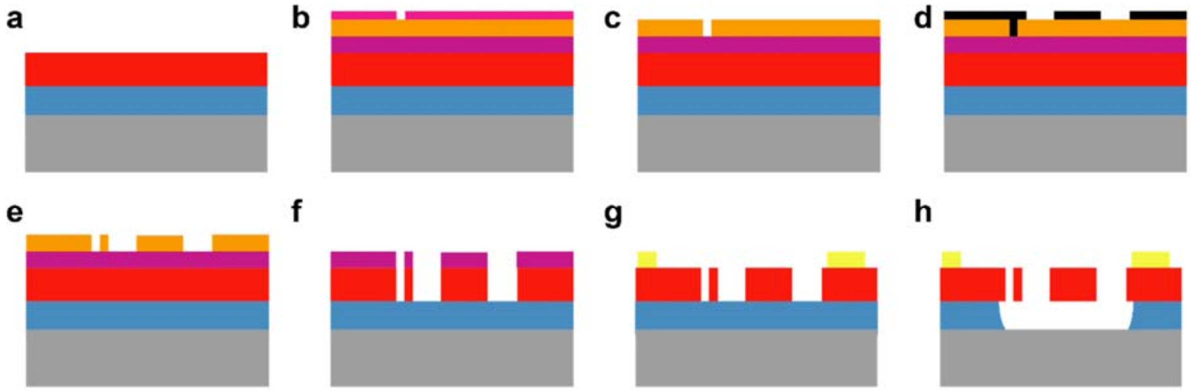

Supplementary Figure S3 Fabrication process for the device (not to scale). **a.** A cross-sectional view of the silicon-on-insulator wafer used in the experiment. From top to bottom, it consists of a silicon device layer (red), a buried oxide layer (blue), and the handle layer (gray). **b**. A layer of thermal oxide is grown on the device layer (purple) and another layer of polysilicon (orange) is then deposited on top. Subsequently, electron-beam lithography is performed to define the patterns of gratings on the electron-beam resist (pink) coated on the wafer. **c**. The areas of polysilicon without protection from the electron-beam resist are etched away by the RIE process. **d**. Optical lithography is performed to define the large patterns, such as the comb drive and the springs, on the coated optical photoresist (black). **e**. The areas of the polysilicon mask without protection from the optical photoresist are etched away by the RIE process. **f**. The patterns on the polysilicon mask are transferred onto the oxide mask by the RIE process and further transferred to the device layer by the DRIE process. During the DRIE process, the polysilicon layer of the mask is also etched away. Two layers of the mask are necessary here to create a nearly vertical profile in the dry etching process. **g**. 5nm-thick chromium and 150nm-thick gold (yellow) are evaporated as the bonding pads defined by optical lithography. **h**. Hydrofluoric acid is used for undercutting the buried oxide layer underneath for freeing the movable parts and removing the residual oxide mask.

We release the movable parts of the device from the buried oxide using hydrofluoric acid. By controlling the etching time, the fixed parts with a larger area of buried oxide layer underneath (such as the anchors of the springs and the fixed comb) are not released while the movable comb drive and springs with width < 5 μm are suspended (as shown in Fig. S4).

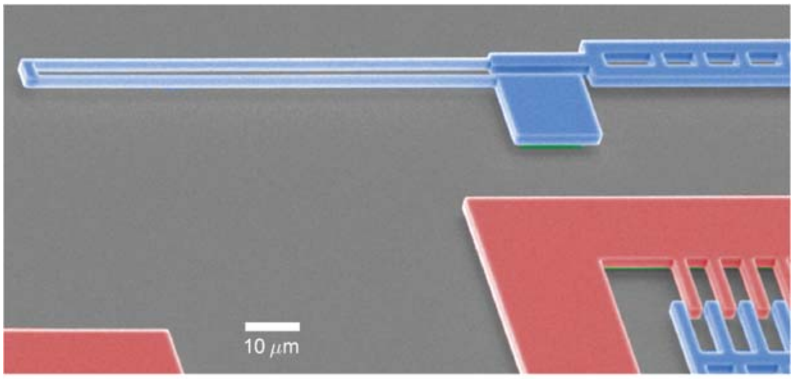

Supplementary Figure S4 False colored micrograph of a spring and part of the comb drives. The red structures are fixed to the substrate via the buried oxide (green) underneath. The blue structures contain thin regions that are suspended because the underlying oxide is removed.

<u>Supplementary Note 4. Improvements from previous experiments using the same monolithic platform</u>

In this work, we made a number of important improvements in the fabrication and design compared to the work presented in Ref. 14. These changes are essential in revealing the strong geometry effect and accessing the interpenetration regime to demonstrate the distance-independent Casimir force. First, we switch from optical lithography to electron-beam lithography to define the gratings as they are the most critical element of the entire structure. It is, however, too time-consuming to define the entire device using electron-beam lithography. Therefore, we continue to use optical lithography for the other parts of the device, including the springs and the comb drives. Second, we add one more mask layer (Poly-Si) on the top of the existing oxide mask to improve

the accuracy of the pattern transfer from the electron beam resist to the device layer. The high selectivity for polysilicon and electron beam resist in the etching process (as compared to that between oxide and resist) improves the accuracy at which the lithographic pattern is transferred. It also allows us to use thinner electron beam resist to define the geometry more accurately. Other changes are also implemented to improve the accuracy of fabrication, such as changing the recipes of etching for better selectivity, adding a cooling step for the electron beam resist and optimizing the electron beam dose distribution based on the proximity effect correction from the Monte-Carlo simulation to achieve the better profiles. Supplementary Figure S5 compares the device used in measurement to an early device fabricated without electron beam lithography. Both structures are nominally designed to be rectangular. Rounding of the corners is much more severe in the latter structure. Moreover, the uniformity among grating units is also much improved from Ref. [14]. For example, the variation of lateral gaps g between the different grating units in this paper is ~90-92 nm while the lateral gap for the top of the T-structures in Ref. [14] varies between 45-90 nm. The latter introduces uncertainty that affects the comparison with theory.

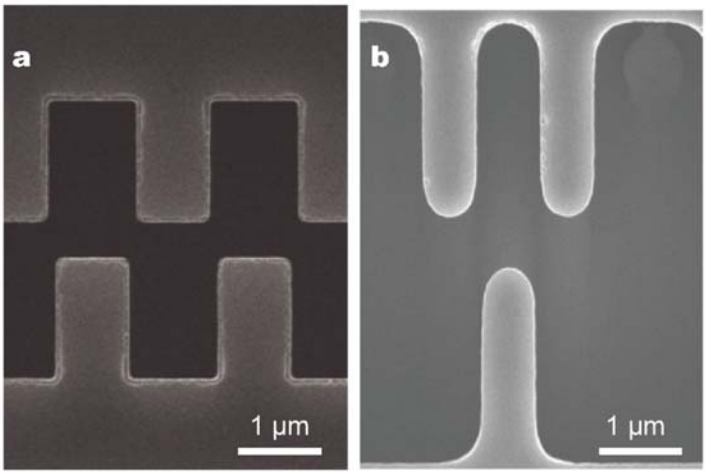

Supplementary Figure S5 Nominally rectangular gratings fabricated by **a** electron beam lithography and the improved process flow in the current paper and **b** optical lithography and the process in Ref. [14]. Much sharper corners and edges are achieved in the current device.

Supplementary Note 5. Algorithm of PAA

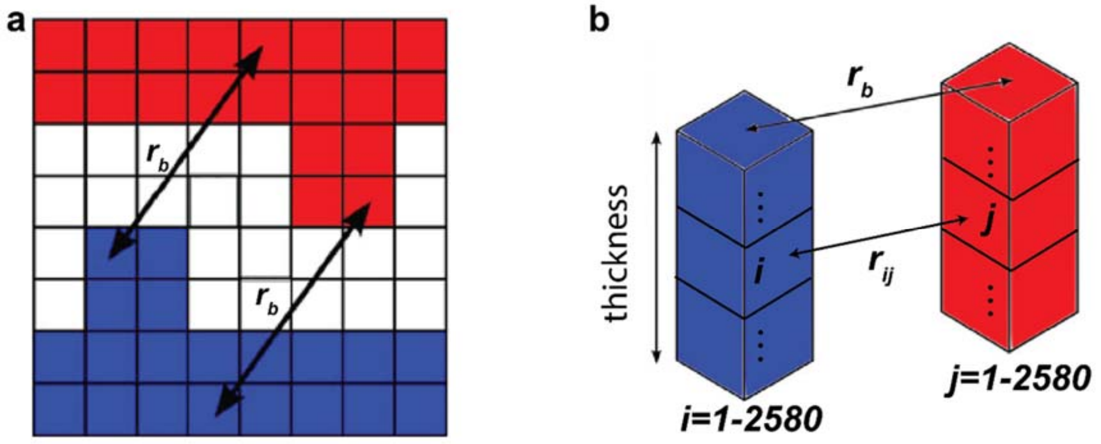

Supplementary Figure S6 Schematic of optimized algorithm for PAA (not to scale). **a** Divide the top view of gratings into pixels that are 1 nm by 1 nm in cross-section and 2580 nm tall. There are two groups of pixels from the two gratings colored in blue and red. **b**. Each pixel is a cuboid containing 2580 cubes of volume 1 nm^3. The van der Waals energy between two cuboids separated by distance r_b is calculated using Eq. (8).

Brute force numerical integration for the pairwise summation expression [Eq. (8)] is computationally highly intensive. As the expression is a double volume integral the spatial integrals alone scale as n^6 where n stands for the number of the basic elements. To improve the calculation speed, the procedure used to calculate U_c is split into three stages.

The first stage takes the top view of the structure (Fig. 1b and Fig. 2b in the main text) and divide it into pixels, as shown in Fig. S6a. Each pixel measures 1 nm by 1 nm in the x-y plane and 2.58 μm in the z-direction. A pixel is further divided along the z-direction into 2580 cubic units, each with a volume of 1 nm^3. The second stage computes the van der Waals' energy of two pixels as a function of their separation r_b by integrating over the frequencies ξ and the thicknesses as shown in Fig. S6b. The procedure yields a data file that lists the van der Waals' energy between two pixels for all the possible separations r_b (10 nm to 10 μm). Since the potential is only a function

of the pixel separation r_b and many pixel pairs have the same separation, the pre-calculated list reduces duplications in the calculation. Thus, the computation time is reduced from n^6 to $n^2 + n^4$, and the repetitions in the calculation of the frequency integral can be avoided. In the last stage, pairs of pixels from the two sides of gratings are identified and their interaction energy is extracted from the list to add up to the total energy U_c.
